# Supplementary figures and images for: Minos-mediated transgenesis in the pantry moth Plodia interpunctella
Source: PeerJ. 2025 Nov 12;13:e20249. doi: 10.7717/peerj.20249 (PMC12619579; doi:10.7717/peerj.20249)

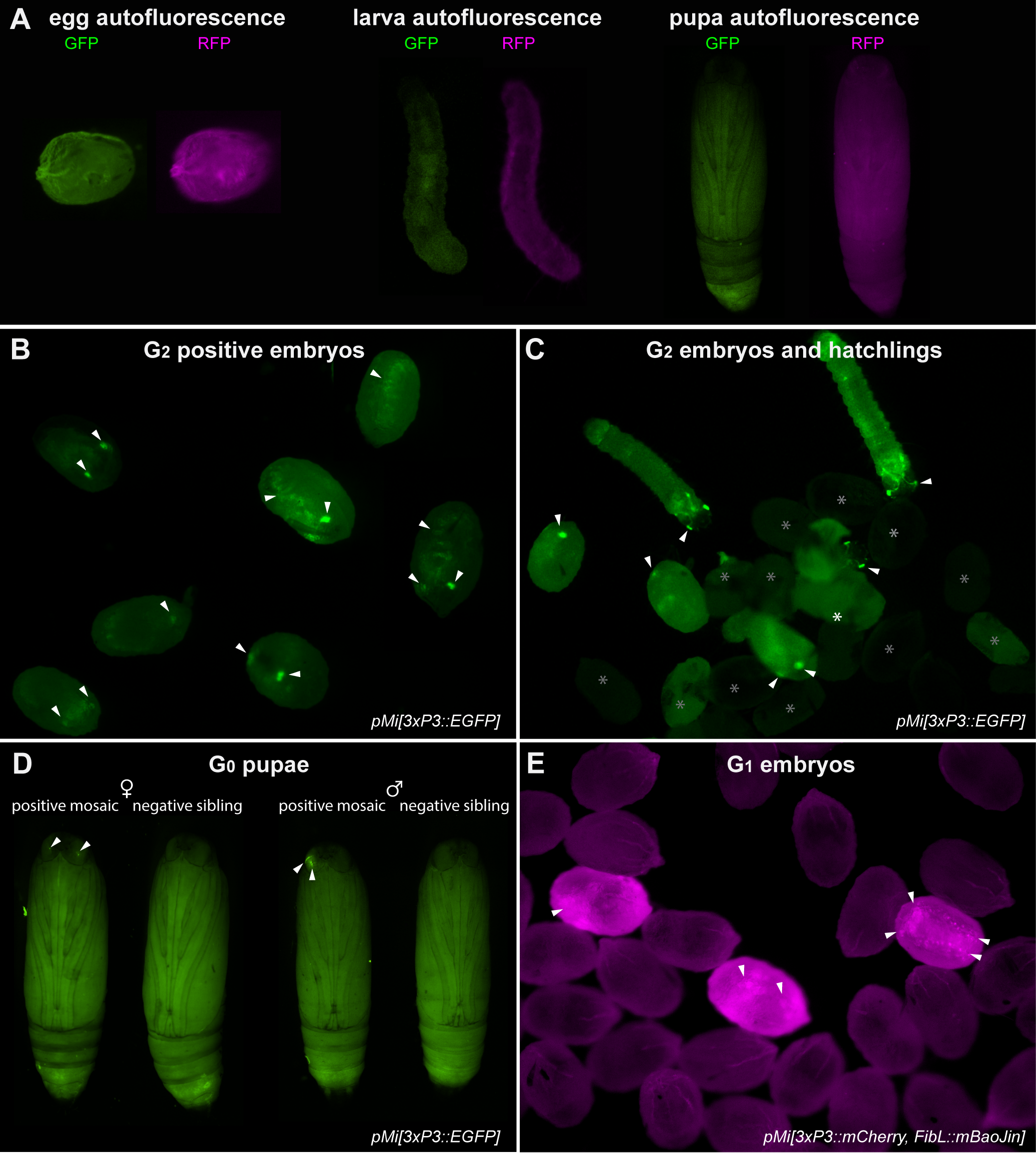

Supplement: Supplemental Information 1 — (A) Autofluorescence of an uninjected control egg (left), larva (middle), and pupa (right), imaged separately under the GFP and RFP filter sets. (B) Examples of G2 embryos positive for 3xP3::EGFP activity in different orientations (from Experiment #1). (C) G2 embryos and hatchlings positive (arrowheads) and negative (asterisks) for 3xP3::EGFP activity (from Experiment #1). (D) Side by side comparison of G0 positive mosaics for 3xP3::EGFP (arrowheads) with their negative siblings, separated by sex. (E) G1 positive (arrowheads) and negative embryos for 3xP3::mCherry (from in-cross of G0 negatives from Experiment #5). Arrowheads (B-E): bright cellular expression of transgene markers not observed in controls [file peerj-13-20249-s001.png]

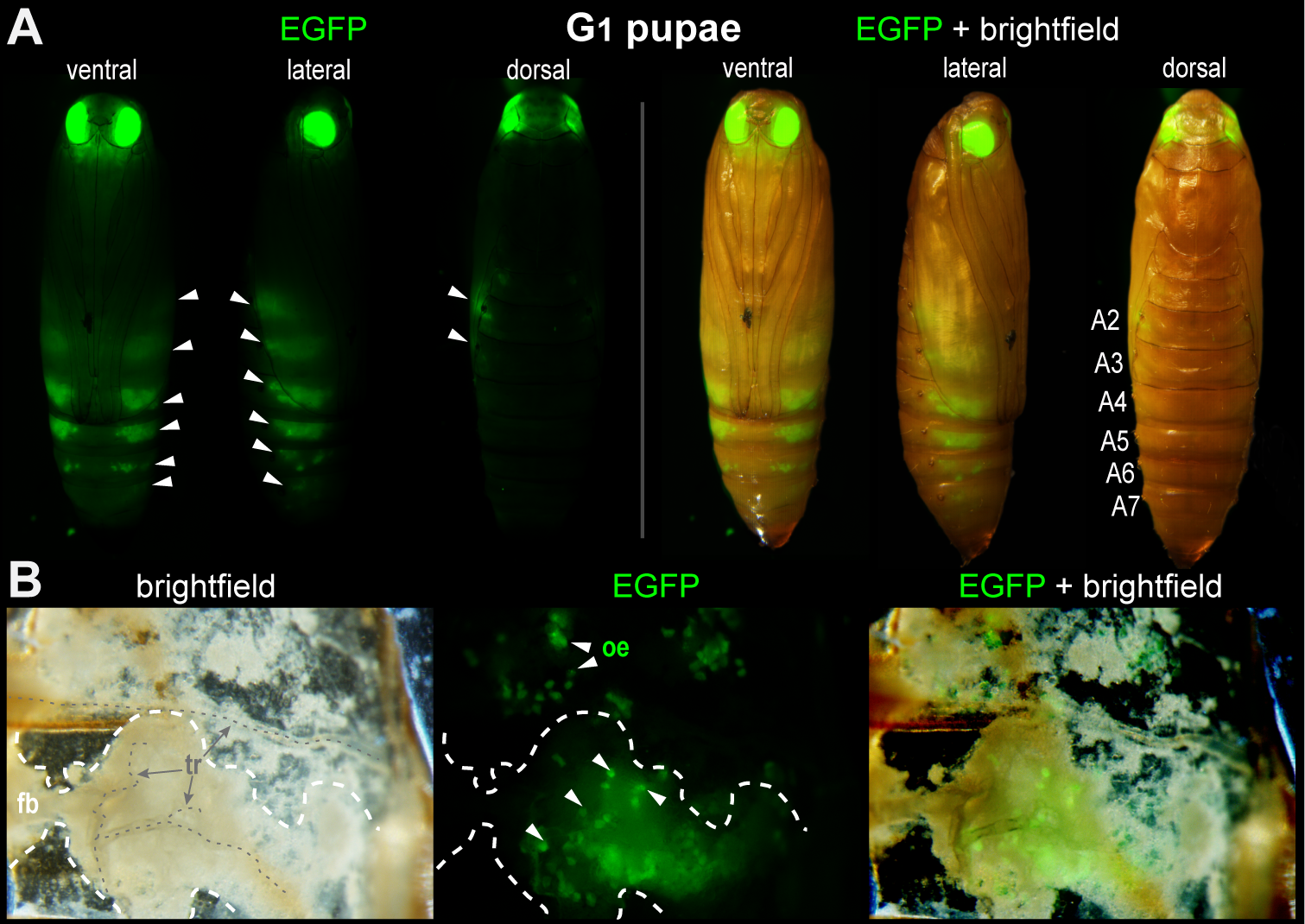

Supplement: Supplemental Information 2 — (A) 10 out of 59 (17%, Experiment #1) G1 pupae positive for 3xP3::EGFP showed fluorescent activity in metameric belts of cells restricted to the abdominal segments A2 to A7 (white arrowheads). This oenocyte-marking activity, likely derived from a specific insertion site, has been maintained over 3 subsequent generations (G2–G4), attesting to the stability of this EGFP+/oeno insertion allele without remobilization. (B) Dissection of the pupal cuticle with internal tissues and fat body partially removed. Fluorescence is detected in large cells embedded under the cuticle and in proximity to tracheal projections. These cells have been described as oenocytes in the closely related phycitine moth Ephestia kuehniella (Stendell, 1912). Abbreviations: tr, trachea ; fb, fat body; oe, oenocytes. [file peerj-13-20249-s002.png]
