# Supplementary material for: Minos-mediated transgenesis in the pantry moth Plodia interpunctella
Source: PeerJ. 2025 Nov 12;13:e20249. doi: 10.7717/peerj.20249 (PMC12619579; doi:10.7717/peerj.20249)
Supplement: Supplemental Information 3 — Mock injections consisted of injections conditions identical to the transgenesis Experiments #1 to #3, minus the presence of transposase mRNA. [file peerj-13-20249-s003.docx]

| **Control experiment** | **Strain** | **Embryos** | | |
| --- | --- | --- | --- | --- |
|  |  | **Injected/collected** | **hatched** | **hatching rate** |
| Mock injection  with *pMi[3xP3::EGFP]*  (no transposase) | wFog (*white* -/-) | 101 | 13 | 12.9% |
|  | wFog (*white* -/-) | 56 | 16 | 28.6% |
|  | **total** | **157** | **29** | **18.5%** |
| Positioned on parafilm, uninjected | wFog (*white* -/-) | 25 | 16 | 64.0% |
|  | wFog (*white* -/-) | 302 | 186 | 61.6% |
|  | **total** | **327** | **202** | **61.8%** |

**Table S1. Control experiments assessing the effect of mock injections on egg hatching rates.** Mock injections consisted of injections conditions identical to the transgenesis Experiments #1 to #3, minus the presence of transposase mRNA.
